# Elevated Troponins and Diagnosis of Non-ST-Elevation Myocardial Infarction in the Emergency Department

**DOI:** 10.7759/cureus.59910

**Published:** 2024-05-08

**Authors:** Farman Ali, Khurram Arshad, Susan Szpunar, Edouard Daher

**Affiliations:** 1 Medicine, Ascension St. John Hospital and Medical Center, Detroit, USA; 2 Internal Medicine, Corewell Health East Dearborn, Dearborn, USA; 3 Biomedical Investigations and Research, Ascension St. John Hospital and Medical Center, Detroit, USA; 4 Cardiology, Ascension St. John Hospital and Medical Center, Detroit, USA

**Keywords:** non-st-segment elevation myocardial infarction (nstemi), acute coronary syndrome (acs), cardiac troponin, troponin, 2d echocardiography, emergency department

## Abstract

Background

In the emergency department (ED), the diagnosis of non-ST-elevation myocardial infarction (NSTEMI) is primarily based on the presence or absence of elevated cardiac troponin levels, ECG changes, and clinical presentation. However, limited data exist regarding the incidence, clinical characteristics, and predictive value of different cardiac diagnostic tests and outcomes in patients with non-acute coronary syndrome (ACS)-related troponin elevation. Our study aimed to determine the percentage of patients with elevated troponin levels who had true ACS and identify various risk factors associated with true ACS in these patients.

Methodology

This was a single-center retrospective study. We performed a chart review of patients who presented to the ED from January 1, 2016, to December 31, 2017, and were admitted to the hospital with an elevated cardiac troponin I level in the first 12 hours after ED presentation with a diagnosis of NSTEMI. True ACS was defined as (a) patients with typical symptoms of ischemia and ECG ischemic changes and (b) patients with atypical symptoms of myocardial ischemia or without symptoms of ischemia and new segmental wall motion abnormalities on echocardiogram or evidence of culprit lesion on angiography. A logistic regression model was used to determine the association between risk factors and true ACS.

Results

A total of 204 patients were included in this study. The mean age of the study group was 67.4 ± 14.5 years; 53.4% (n = 109) were male, and 57.4% (n = 117) were Caucasian. In our study, 51% of patients were found to have true ACS, and the remaining 49% had a non-ACS-related elevation in troponins. Most patients without ACS had alternate explanations for elevated troponin levels. The presence of chest pain (odds ratio (OR) = 3.7, 95% confidence interval (CI) = 1.8-7.7, p = 0.001), tobacco smoking (OR = 4, 95% CI = 1.06-3.8, p = 0.032), and wall motion abnormalities on echocardiogram (OR = 3.8, 95% CI = 1.8-6.5, p = 001) were associated with increased risk of true ACS in patients with elevated troponins.

Conclusions

Cardiac troponin levels can be elevated in hospitalized patients with various medical conditions, in the absence of ACS. The diagnosis of ACS should not be solely based on elevated troponin levels, as it can lead to expensive workup and utilization of hospital resources.

## Introduction

Chest pain is the second most common complaint during emergency department (ED) visits [1]. Approximately 10 million annual ED visits in the United States are due to chest pain [2, 3]. Acute coronary syndrome (ACS) includes ST-elevation myocardial infarction (STEMI), non-ST-elevation myocardial infarction (NSTEMI), and unstable angina (UA). Risk factors for ACS include hypertension, diabetes mellitus, smoking, male sex, hyperlipidemia, advanced age, prior history of coronary artery disease (CAD), and family history of premature CAD [4]. In the United States, CAD is the leading cause of mortality [5]. STEMI is diagnosed based on ST-segment elevation in two contiguous leads on ECG in appropriate clinical settings [6]. NSTEMI and UA represent a spectrum of pathologies that involve an imbalance in oxygen supply and demand available to the cardiac muscles [7]. UA and NSTEMI are differentiated by the presence or absence of elevated cardiac troponin (cTn) levels, which signify myocardial damage [8]. Among patients diagnosed with ACS, 70% have NSTEMI diagnosed using cTn assays [9]. cTn is a protein present in the contractile apparatus of cardiomyocytes. They are released into the blood after myocardial injury. There are two subtypes of cTn: cardiac troponin T (cTnT) and cardiac troponin I (cTnI) [10]. Owing to their tissue specificity, cTnT and cTnI assays are the principal blood tests used to diagnose ACS or other conditions when myocyte damage is suspected. Hence, they are commonly used in screening blood tests in the healthcare setting [11].

One of the main goals of ED evaluation for patients presenting with chest pain or other equivalent anginal symptoms (e.g., chest discomfort, indigestion, and shortness of breath) is to rule out ACS. Although chest pain is usually the primary symptom of ACS, diagnosing it correctly, especially in the ED, can be challenging, as many patients do not present with typical ACS symptoms.

Detecting cTn is not pathognomonic for diagnosing ACS; it indicates cardiac muscle injury from any cause and can be elevated in many non-coronary artery-related conditions [7]. Elevated troponin levels can be due to cell damage (either reversible or irreversible), increased membrane permeability, reversible oxygen deficits, or non-cardiac etiologies such as kidney disease, congestive heart failure (CHF), pulmonary embolism (PE), or sepsis [12].

In the ED, the diagnosis of NSTEMI is primarily based on elevated troponin levels when the clinical presentation is unclear. The decision to initiate dual antiplatelet therapy (DAPT) in patients with NSTEMI is crucial and has been shown to improve the prognosis. Some prefer to treat all patients with NSTEMI with DAPT [13]. Owing to various factors, troponin levels are over-utilized and over-ordered in busy ED settings, including in patients who may not have apparent clinical symptoms and signs suggestive of ACS. Given that DAPT can be lifesaving in true ACS and injurious outside of it, it is essential to determine the significance of elevated troponins, whether due to coronary-related cardiac disease or other causes.

This retrospective study aimed to investigate the percentage of patients with true ACS upon discharge following an initial diagnosis of NSTEMI and factors associated with cardiac ischemia.

## Materials and methods

This study was approved by the Institutional Review Board of Ascension St. John Hospital, Detroit, MI, USA (approval number: 1173141). A chart review was performed retrospectively on adult patients admitted to St. John Hospital with an initial diagnosis of NSTEMI between January 1, 2016, and December 31, 2017. Adult patients with elevated cTnI levels within the first 12 hours after presentation to the ED and diagnosed with NSTEMI were included. Patients with STEMI and UA were excluded from this study. Baseline information included age, race, sex, length of hospital stay, chest pain on presentation, troponin levels, renal function stage, history of smoking, hypertension, diabetes mellitus, hyperlipidemia, prior CAD, cardiovascular accident, discharge diagnosis, echocardiography, stress test, and cardiac catheterization. cTn levels were measured using an Abbott Axym system (Abbott Laboratories, Mississauga, Ontario, Canada). An increase in troponin I level was defined as a serum level >2 ng/mL, as recommended by the manufacturer.

The ACS group represents patients who develop acute myocardial infarction believed to be caused by thrombotic or thromboembolic phenomena, leading to significantly altered blood flow in the coronary arteries. The following criteria were applied to diagnose ACS-related increase in troponin levels: (A) patients with typical symptoms of ischemia and ECG ischemic changes (new ST-segment depression, T-wave inversion) and (B) patients with atypical symptoms of myocardial ischemia or without symptoms of ischemia were diagnosed with ACS as the cause of elevated troponins if they had new segmental wall motion abnormalities on echocardiogram or evidence of culprit lesions on angiography. Those with elevated cTnI levels and no wall motion abnormalities seen on echocardiogram or normal angiography were considered the non-ACS group.

Sample size

It has been reported that among patients admitted with elevated troponin, 65% have ACS [14]. To find a similar percentage in our study group with an alpha error rate of 0.05 and 80% power, a total of 204 patients need to be studied, 102 in each group.

Statistical analysis

Continuous variables were presented as mean (±standard deviation) or median and interquartile range, while categorical variables were described using frequency distributions. The chi-square test was used to compare categorical variables, and the Student’s t-test was used to compare continuous variables. Multivariate analysis was performed using logistic regression with variables selected based on backward selection, investigator preference, and prior literature. Data were analyzed using SPSS version 25.0 (IBM Corp., Armonk, NY, USA), and a p-value of 0.05 or less was considered to indicate statistical significance.

## Results

In total, 204 patients were included in this study. The mean age was 67.4 ± 14.5 years; 53.4% (n = 109) were male, and 57.4% (n = 117) were Caucasian. Of the total, 180 (88.2%) had a history of hypertension, 180 (88.2%) had chest pain upon presentation, 69 (33.8%) were smokers, 58 (28.6%) had diabetes mellitus, and 58 (28.6%) had a history of CAD. An echocardiogram was performed on 168 (82.4%) patients; among those, 60 (36.3%) had segmental wall motion abnormalities. Cardiac catheterization was performed in 149 (73.0%), and four (3.0%) patients underwent cardiac stress testing. Of the 204 patients, 104 (51%) were found to have true ACS caused by primary cardiac ischemia, and the remaining 100 (49%) patients were considered to have non-ACS-related troponin increase (Table 1).

**Table 1:** Baseline characteristics of the study population. ACS = acute coronary syndrome; CAD = coronary artery disease; CVA = cerebrovascular accident; IQR = interquartile range

Characteristic	ACS group (%)	Non-ACS group (%)	P-value
Age	67 +/13.5	67+/ 15.4	0.40
Race (White)	55.6	44.4	0.13
Sex (male)	53.2	46.2	0.50
Chest pain	58.7	41.3	<0.0001
Smoking	63.8	36.2	0.009
Hyperlipidemia	55.1	44.9	0.09
Hypertension	53.3	46.7	0.06
Diabetes mellitus	56.9	43.1	0.30
History of CAD	54.4	45.6	0.43
History of CVA	55	45	0.70
Wall motion abnormalities on echocardiogram	67.6	32.4	<0.0001
Troponin levels (median, IQR)	0.27 (0.03, 11.95)	0.25 (0.05, 3.38)	0.35
Length of stay (median, IQR)	3.0 (1.0, 27)	3.0 (0.5, 50)	

Of 104 patients with non-ACS, 31 (30%) had acute heart failure, 20 (19%) were diagnosed with sepsis, 19 (18%) had stable CAD, 12 (10%) had acute or acute chronic kidney disease, seven (7%) had myocarditis, five (5%) had anemia, four (4%) had atrial or ventricular arrhythmias, four (4%) had uncontrolled hypertension, and two (2%) patients had another diagnosis such as diabetic ketoacidosis and hypoxemia.

Factors associated with ACS

A multivariable model showed that chest pain (odds ratio (OR) = 3.7, 95% confidence interval (CI) 1.8-7.7, p = 0.001), tobacco smoking (OR = 3.5, 95% CI = 1.06-3.8, p = 0.032), and wall motion abnormalities on echocardiogram (OR = 3.8, 95% CI = 1.8-6.5, p = 001) were associated with ACS. Patients with true ACS had a higher percentage of chest pain, smoking history, and wall motion abnormalities on echocardiography. The history of hypertension, hyperlipidemia, diabetes mellitus, previous CAD, and stroke was similar in both groups. The ACS and non-ACS groups had similar lengths of stay (Table 2).

**Table 2:** Multivariable model showing factors associated with acute coronary syndrome. OR = odds ratio; CI = confidence interval

Characteristics	OR (95% CI)	P-value
Chest pain	3.7 (1.8–7.7)	0.0001
Smoking	4 (1.06–3.8)	0.032
Wall motion abnormality	3.4 (1.8–6.5)	0.0001
Hypertension	2.4 (0.89–6.3)	0.084

## Discussion

cTns are commonly used in patients with symptoms concerning for ACS [15]. Our study showed that only 51% of patients had true ACS. The presence of chest pain, history of smoking, and wall motion abnormalities on echocardiography were associated with true ACS. All patients in the non-ACS group had a preexisting medical condition that had previously been associated with an increase in troponin levels in the absence of ACS.

cTn is a sensitive and specific biomarker of myocardial injury, with elevated troponin levels being associated with a higher risk of death and re-infarction in patients presenting with NSTEMI [16-19]. However, elevated cTnI levels are not a specific diagnostic test for ACS [20]. Various studies have reported elevated troponin levels in many conditions (cardiac or non-cardiac) in the absence of true ACS. Additionally, several studies have linked elevated troponin levels in non-ACS patients to poor outcomes and increased mortality rates [14,21,22].

The elevation in troponin level can be classified as follows: (a) primary ischemic cardiac injury caused by plaque rupture or intraluminal thrombus (e.g., acute myocardial infarction); (b) secondary ischemic cardiac injury caused by myocardial oxygen demand and supply mismatch (e.g., tachy/brady arrhythmias, severe respiratory failure, severe anemia, coronary endothelial dysfunction, spasm, or dissection); (c) non-ischemic cardiac injury caused by damage to myocytes (e.g., cardiac contusion, surgery, ablation, pacing or defibrillation, myocarditis, and cardiotoxic agents such as anthracyclines); (d) Multifactorial or indeterminate myocardial injury with no clear cardiac damage mechanism (heart failure, stress cardiomyopathy, pulmonary embolism, sepsis, critical illness, renal failure, and severe acute neurological disease such as stroke and subarachnoid hemorrhage) [23]. In patients with heart failure, several mechanisms lead to an increase in troponin levels, including subendocardial ischemia and necrosis, ongoing cell death induced by myocardial stretch, and toxic cytokines [24,25]. Many conditions that affect myocardial cell metabolism (carbon monoxide intoxication, hypoxemia, ketoacidosis, hypercarbia, and acute bleeding) can lead to an increase in serum troponin levels [26-28].

cTn is frequently ordered in patients who present to the ED with chest pain, other symptoms of ACS, and various other conditions in which myocyte damage is suspected. Elevated cTn levels have prognostic value in the absence of true ACS [29]. While elevated cTn levels are of diagnostic and prognostic significance, they can frequently lead to the overdiagnosis of NSTEMI in the ED. Many clinicians rely solely on cTn elevation to diagnose NSTEMI when the clinical presentation is unclear.

Elevated cTn level caused by primary cardiac ischemia or another mechanism is of paramount therapeutic importance. Patients with elevated troponin levels without true ACS have a high mortality rate and should be started on appropriate therapy aimed at the underlying cause. However, misdiagnosis can lead to unnecessary antithrombotic therapy, which may be inappropriate for these patients. This can result in increased hospitalization costs and overutilization of healthcare resources, underscoring the importance of an accurate diagnosis.

One of the studies conducted by Blich et al. found that with an initial diagnosis of ACS, only 65% of patients were found to have true ACS, and the remaining 35% had non-ACS-related troponin increase [14]. In another study of 69,299 patients who were admitted from the ED, 33,263 (48%) individuals underwent testing for cTn levels. Of these, 2,344 patients (3.3% overall or 7.0% of those tested for cTn) were found to have elevated cTn levels. Notably, 42.7% of patients with a positive cTn result did not have ACS [29].

In our study, only 51% (n = 104) of patients were found to have true ACS caused by primary cardiac ischemia, while the remaining 49% (n = 100) had elevated troponin levels due to other known medical conditions. The magnitude of the increase in troponin level was not helpful in diagnosing true ACS. The presence of elevated troponin levels in hospitalized patients presents a diagnostic challenge.

In our study, an alternative diagnosis explaining the finding of increased troponin level was present in almost all patients when the initial clinical presentation was inconsistent with true ACS. The most common cause of cTn elevation in the non-ACS group was heart failure; other causes included sepsis, acute kidney injury, chronic kidney disease, atrial fibrillation with rapid ventricular rate, myocarditis, hypertensive emergency, and anemia. Table 3 highlights the differential diagnosis of elevated cTn according to various etiologies of myocyte damage [30].

**Table 3:** Causes of elevated troponins. ACS = acute coronary syndrome; AMI = acute myocardial infarction, ESRD = end-stage renal disease; HF = heart failure; NSTEMI = non-ST-elevation myocardial infarction; PCI = percutaneous coronary intervention; STEMI = ST-elevation myocardial infarction; cTnI = cardiac troponin I; cTnT = cardiac troponin T; ROC = receiver operating characteristic

Troponin elevation (disease)	Prevalence of troponins (cut-offs)	Mechanism of troponin release
ACS related		
AMI	100% per definition	Thrombotic occlusion of coronary artery (STEMI), microembolization (NSTEMI)
Post-PCI	31% (cTnI) – 40% (cTnI); 24% (cTnT)	Side branch occlusion, coronary dissection, bulky devices causing transient ischemia and micro embolisms
Open heart surgery	100% (cTn T)	Myocardial infarction, incomplete cardioprotection, reperfusion injury, direct surgical trauma
Non-ACS-related		
Acute pulmonary embolism	Variable, depending on cut-off; 32% at cTnT >0.1 ng/mL – 50% at cTnT >0.01 ng/mL	Right ventricular strain
Asymptomatic patients with ESRD	Variable, depending on cut-off; 99th centile/10% CV/ROC: 82%/53%/20% for cTnT and 6%/1%/0.4% for cTnI	Several possible reasons including coronary and non-coronary cardiac origin; prolonged renal elimination; non-dialysable, intact cTnT; and differences to cTnI may be related to higher affinity to dialysis membrane
Pericarditis/myocarditis	32–49% (cTnI)	Direct damage to myocytes
Aortic dissection Stanford A	24% (cTnI >1.5 ng/mL)	Dissection of coronary artery
Chronic HF	15% (cTnT >0.1 ng/ml) –23% (stable and unstable) (cTnI >0.3 ng/mL)	Global wall stretch, degradation of contractile protein, and cellular injury due to oxidative stress and neurohumoral factors
Acute HF	52% (cTnT ≥0.02 ng/ml) –55% (cTnT ≥0.1 ng/mL)	Global wall stretch, hypoxemia, systemic hypoperfusion, coronary malperfusion
Strenuous exercise/ultra-endurance athletes	26% (cTnT), 9% (cTnI); 23% (cTnT), 32% (cTnI)	Ventricular stretch, release of soluble troponin, underlying cardiac disease
Cardiotoxic chemotherapy	Unknown	Direct toxic effect on myocytes
High-frequency ablation/ current cardioversion-defibrillator shocks	90% (cTnI)	Direct myocardial damage
Cardiac infiltrative disorders (amyloidosis)	Unknown	Myocyte compression
Heart transplant	100% (up to 3 months) variable after 3 months	Inflammatory/immune-mediated
Cardiac contusion after blunt chest wall trauma	12% (cTnI) – 15% (cTnT)	Direct myocardial damage
Sepsis/critically ill patients	36% (cTnT ≥0.1 ng/ml) – 85% (cTnT >0.1 ng/mL)	Oxygen supply/demand mismatch, cytokine/endotoxin mediated toxicity, heterophilic antibodies (false positive)
Rhabdomyolysis	Unknown	Cross-reactivity between skeletal and cardiac muscle isoforms of troponins in first and second-generation troponin assays

With the development of the high-sensitivity troponin assay, the diagnosis of NSTEMI has become easier and quicker. Many institutions in the United States are adopting it, leading to the detection of many patients with increased troponin levels. Clinicians should familiarize themselves with the broad differential diagnosis of elevated cTn to prevent the misdiagnosis and treatment of presumed ACS, avoiding delays in the treatment of the underlying condition.

Our study indicates that 49% of selected patients undergoing troponin testing at a community hospital have increased troponin levels that were likely unrelated to ACS. Future studies are needed to further investigate the mechanism of elevated troponin levels caused by true cardiac ischemia or other etiologies. Relying solely on serum troponin levels to diagnose NSTEMI when the clinical presentation is inconsistent with ACS is insufficient, and clinicians should also consider other conditions in the differential diagnosis. Clinicians need to be aware that cTn is not only a biomarker of ischemic myocardial injury but can also be elevated outside of ACS.

Study limitations

As this was a single-center retrospective observational study, there is a risk of selection bias. Owing to the small study population, statistical analysis has an inherent risk of beta error. Furthermore, the duration of the symptom onset is unknown. Due to time constraints, we could not calculate each group’s one-year mortality rate for outcome evaluation.

## Conclusions

The diagnosis of NSTEMI should not be based solely on the presence of elevated cTn levels. Many cardiac, pulmonary, and systemic diseases can present with elevated cTn outside of ACS, reflecting cardiac myocyte necrosis.

An elevated cTn level in the absence of ACS carries a worse prognosis, and these conditions should be managed appropriately without delaying appropriate treatment.

The increased use of high-sensitivity troponin assays will result in more frequent detection of elevated cTn, and clinicians should familiarize themselves with the broad differential diagnosis of elevated cTns to avoid unnecessary misdiagnosis and treatment of presumed ACS.
